# FGF19 functions as autocrine growth factor for hepatoblastoma

**DOI:** 10.18632/genesandcancer.101

**Published:** 2016-03

**Authors:** David J. Elzi, Meihua Song, Barron Blackman, Susan T. Weintraub, Dolores López-Terrada, Yidong Chen, Gail E. Tomlinson, Yuzuru Shiio

**Affiliations:** ^1^ Greehey Children's Cancer Research Institute, The University of Texas Health Science Center, San Antonio, Texas, USA; ^2^ Department of Biochemistry, The University of Texas Health Science Center, San Antonio, Texas, USA; ^3^ Department of Epidemiology and Biostatistics, The University of Texas Health Science Center, San Antonio, Texas, USA; ^4^ Department of Pediatrics, The University of Texas Health Science Center, San Antonio, Texas, USA; ^5^ Department of Pathology and Pediatrics, Texas Children's Hospital and Baylor College of Medicine, Houston, Texas, USA

**Keywords:** cytokine, FGF19, hepatoblastoma, proteomics, secretome

## Abstract

Hepatoblastoma is the most common liver cancer in children, accounting for over 65% of all childhood liver malignancies. Hepatoblastoma is distinct from adult liver cancer in that it is not associated with hepatitis virus infection, cirrhosis, or other underlying liver pathology. The paucity of appropriate cell and animal models has been hampering the mechanistic understanding of hepatoblastoma pathogenesis. Consequently, there is no molecularly targeted therapy for hepatoblastoma.

To gain insight into cytokine signaling in hepatoblastoma, we employed mass spectrometry to analyze the proteins secreted from Hep293TT hepatoblastoma cell line we established and identified the specific secretion of fibroblast growth factor 19 (FGF19), a growth factor for liver cells. We determined that silencing FGF19 by shRNAs or neutralizing secreted FGF19 by anti-FGF19 antibody inhibits the proliferation of hepatoblastoma cells. Furthermore, blocking FGF19 signaling by an FGF receptor kinase inhibitor suppressed hepatoblastoma growth. RNA expression analysis in hepatoblastoma tumors revealed that the high expression of FGF19 signaling pathway components as well as the low expression of FGF19 signaling repression targets correlates with the aggressiveness of the tumors. These results suggest the role of FGF19 as autocrine growth factor for hepatoblastoma.

## INTRODUCTION

Hepatoblastoma is the most common malignant liver tumor in children and accounts for over 65% of all childhood liver malignancies [1]. While the survival rate is high if hepatoblastoma tumor can be completely resected, the prognosis of unresectable metastatic cases continues to be poor [2]. Unlike adult liver cancers, hepatoblastoma is not associated with hepatitis virus infection, cirrhosis, or other underlying liver pathology [3]. There are only a very small number of hepatoblastoma cell lines, which has been the major obstacle to understanding the mechanism of hepatoblastomagenesis and evaluating new therapeutic approaches.

We have previously established a valuable cell model for hepatoblastoma, Hep293TT, from surgically resected hepatoblastoma tissue of a 5-year-old Caucasian female patient [4]. To dissect the cytokine signaling in hepatoblastoma, we used secretome proteomics to analyze the proteins secreted from Hep293TT cells and identified the specific secretion of FGF19.

FGF19 functions as a growth factor for liver cells [5, 6]. FGF19 gene amplification was shown to act as a driver for adult hepatocellular carcinoma [7]. However, the role of FGF19 in childhood hepatoblastoma is unknown. Importantly, our data indicate that silencing FGF19 signaling by FGF19 shRNAs, anti-FGF19 neutralizing antibody, or an FGF receptor kinase inhibitor, LY2874455, inhibits the growth of hepatoblastoma cells, suggesting that FGF19 is a critical autocrine growth factor for hepatoblastoma. Furthermore, the gene expression analysis of hepatoblastoma tumors uncovered that the high expression of FGF19 signaling pathway components as well as the low expression of FGF19 signaling repression targets correlates with the aggressiveness of the tumors. These results suggest that hepatoblastoma is dependent on autocrine secretion of FGF19, which can be targeted therapeutically.

## RESULTS AND DISCUSSION

### Secretome proteomic analysis of Hep293TT hepatoblastoma cells

**Figure 1:**
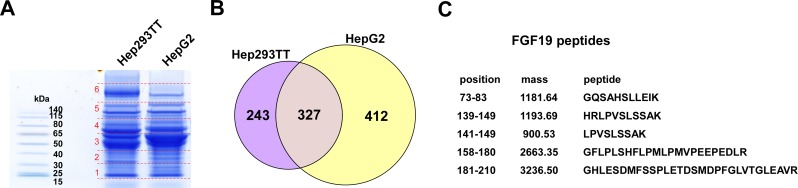
Secretome proteomic analysis of Hep293TT hepatoblastoma cells **A.** GeLC-MS analysis of proteins secreted from Hep293TT and HepG2 cells. Thirty μg each of the secreted proteins were fractionated by SDS-PAGE and each lane was divided into six slices. The proteins in each gel slice were digested with trypsin and were analyzed by liquid chromatography - tandem mass spectrometry. **B.** A Venn diagram indicating the number of proteins commonly and uniquely identified in the Hep293TT and HepG2 secretome. **C.** FGF19 peptides identified with high confidence in the Hep293TT secretome.

To dissect the cytokine signaling in hepatoblastoma, we analyzed the proteins secreted from Hep293TT hepatoblastoma cells by mass spectrometry in comparison with HepG2, a widely used liver cancer cell line. Thirty μg each of the secreted proteins were fractionated by SDS-PAGE and each lane was divided into six slices (Figure 1A). The proteins in each slice were in-gel digested with trypsin, analyzed by liquid chromatography - tandem mass spectrometry, and were identified by searching the human IPI protein database with X! tandem. At a Protein Prophet probability score of 0.9 or higher, 570 and 739 proteins were identified in the Hep293TT and HepG2 secretome, respectively. Of these, 327 proteins were commonly identified in the Hep293TT and HepG2 secretome (Figure 1B), which is consistent with the same tissue of origin for these two cancer cell lines. Complete lists of identified proteins are provided in Supporting Information, Table S1 and S2. This analysis identified 5 unique and 6 total peptides derived from FGF19 in the Hep293TT secretome (Figure 1C, Supplementary Table S1). FGF19 secretion was not detected in the HepG2 secretome (Supplementary Table S2) or in the secretome of several other childhood cancers including osteosarcoma, Ewing sarcoma, neuroblastoma, rhabdomyosarcoma, and retinoblastoma (not shown). Quantitative RT-PCR demonstrated that Hep293TT cells express much higher levels of FGF19 mRNA than HepG2: Hep293TT 4.19 ± 0.17 *vs*. HepG2 0.00118 ± 0.00043 (FGF19 mRNA levels normalized against RNA polymerase II mRNA levels).

### Hepatoblastoma is dependent on autocrine secretion of FGF19

FGF19 is a growth factor for liver cells [5, 6]. FGF19 is mitogenic to hepatocytes [8] and muscle-specific FGF19 transgenic mice develop liver cancer by 10 months of age [9]. The FGF19 gene is amplified in about 15% of adult hepatocellular carcinoma and is considered a driver of this cancer type [7]. Targeting FGF19 by shRNA-mediated knockdown or by anti-FGF19 antibody neutralization was shown to inhibit the clonogenicity and tumorigenicity of FGF19-amlified hepatocellular carcinoma, which led to a proposal that anti-FGF19 therapy is a promising approach to treat adult hepatocellular carcinoma [7].

**Figure 2:**
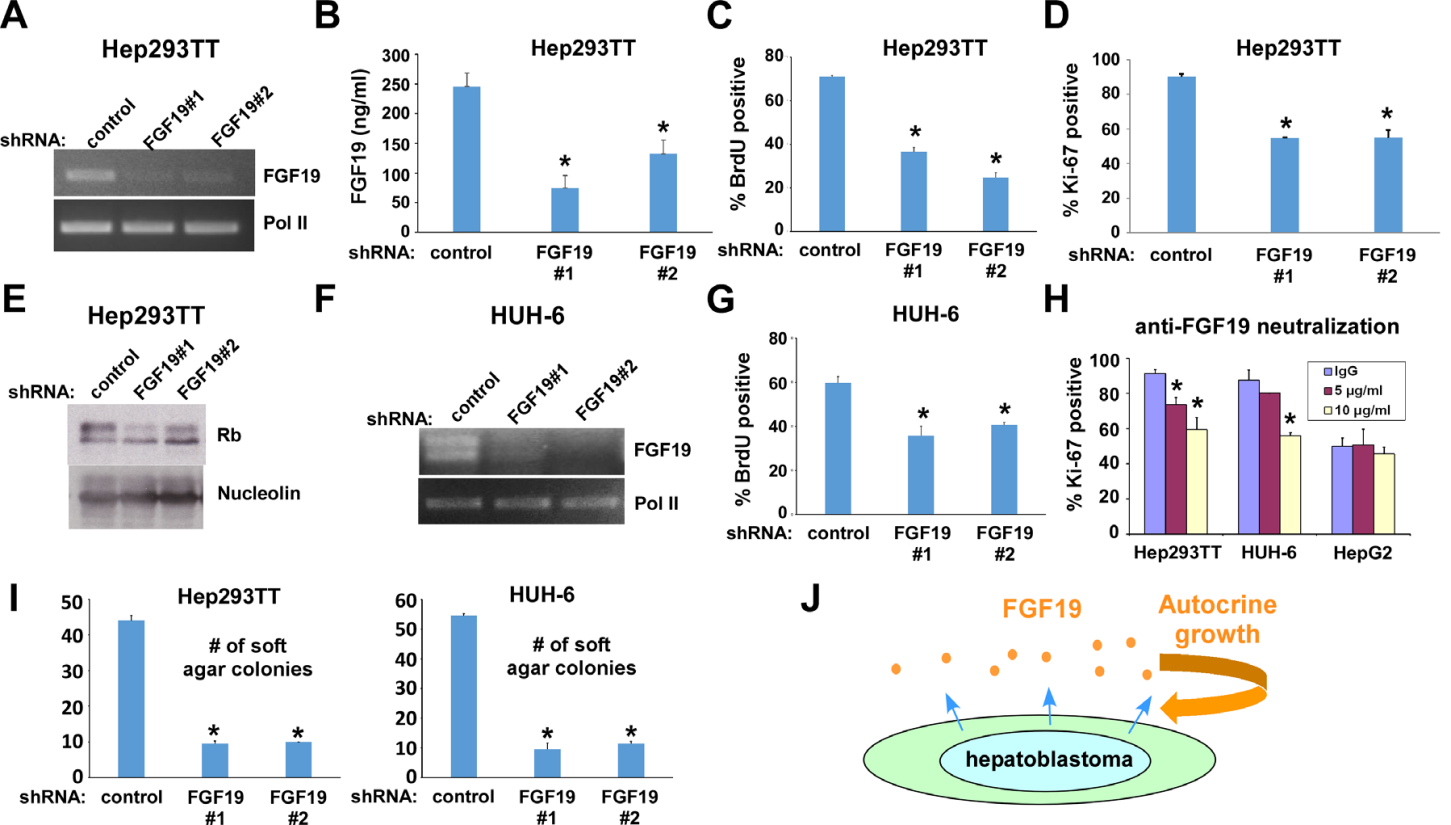
FGF19 downregulation results in inhibition of hepatoblastoma proliferation **A.** shRNA-mediated silencing of FGF19 mRNA expression in Hep293TT cells. Hep293TT cells were infected with lentiviruses expressing shRNAs against FGF19 or control shRNA and were selected with 2 μg/ml puromycin. Four days after infection, total RNA was isolated and the expression of FGF19 was analyzed by RT-PCR. RNA polymerase II (Pol II) serves as a loading control. **B.** Suppression of secreted FGF19 protein levels by shRNAs. Hep293TT cells were infected with lentiviruses expressing shRNAs against FGF19 or control shRNA and were selected with 2 μg/ml puromycin. Four days after infection, the levels of secreted FGF19 protein were assessed by ELISA. **C.** - **E.** FGF19 silencing inhibits Hep293TT cell proliferation. Hep293TT cells were infected with lentiviruses expressing shRNAs against FGF19 or control shRNA. Four days after infection, cells were labeled with BrdU for 6 hours and immunofluorescence microscopy was used to score the percentage of BrdU-positive cells (C); cells were stained for Ki-67 (D); and the dephosphorylation of Rb was analyzed by immunoblotting (E; nucleolin serves as a loading control). Asterisks denote *p* < 0.05 compared with control shRNA. **F.** FGF19 silencing in HUH-6 cells. HUH-6 cells were infected with lentiviruses expressing shRNAs against FGF19 or control shRNA and were selected with 2 μg/ml puromycin. Four days after infection, total RNA was isolated and the expression of FGF19 was analyzed by RT-PCR. RNA polymerase II (Pol II) serves as a loading control. **G.** FGF19 silencing inhibits HUH-6 cell proliferation. HUH-6 cells were infected with lentiviruses expressing shRNAs against FGF19 or control shRNA. Four days after infection, cells were labeled with BrdU for 6 hours. Immunofluorescence microscopy was used to score the percentage of BrdU-positive cells. Asterisks denote *p* < 0.05 compared with control shRNA. **H.** FGF19 antibody neutralization inhibits hepatoblastoma cell proliferation. Hep293TT, HUH-6, and HepG2 cells were treated with 10 μg/ml mouse IgG (control), or 5 μg/ml and 10 μg/ml anti-FGF19 antibody for 24 hours. Cells were stained with anti-Ki-67 antibody, and immunofluorescence microscopy was used to determine the percentage of Ki-67-positive cells. Asterisks denote *p* < 0.05 compared with IgG control. **I.** FGF19 silencing impairs anchorage-independent growth of hepatoblastoma cells. Hep293TT and HUH-6 cells were infected with lentiviruses expressing shRNAs against FGF19 or control shRNA and were selected with 2 μg/ml puromycin. Four days after infection, cells were plated in semi-solid medium. One week after culture, colonies were counted. Asterisks denote *p* < 0.05 compared with control shRNA. **J.** FGF19 functions as an autocrine growth factor for hepatoblastoma.

To address the role of FGF19 in childhood hepatoblastoma, we used lentivirus to express shRNAs against FGF19 in Hep293TT hepatoblastoma cells, which resulted in reduced FGF19 transcript levels as determined by RT-PCR (Figure 2A, RNA polymerase II [Pol II] serves as a control), reduced FGF19 secretion as determined by ELISA (Figure 2B), and reduced cell proliferation as assessed by BrdU incorporation (Figure 2C), Ki-67 staining (Figure 2D), and dephosphorylation of Rb (Figure 2E). We obtained a second hepatoblastoma cell line (HUH-6) derived from a Japanese male infant from JCRB Cell Bank. Silencing FGF19 in HUH-6 hepatoblastoma cells (Figure 2F) also inhibited cell proliferation (Figure 2G).

Furthermore, neutralization of secreted FGF19 by anti-FGF19 antibody resulted in dose-dependent inhibition of Hep293TT and HUH-6 cell proliferation (Figure 2H; IgG control, 5 μg/ml, or 10 μg/ml anti-FGF19). HepG2 cells, which were previously shown to be insensitive to anti-FGF19 neutralization [7], were not growth inhibited by anti-FGF19 antibody (Figure 2H).

In addition, we tested the role of FGF19 in anchorage-independent growth of hepatoblastoma by soft agar colony formation assays. As shown in Figure 2I, FGF19 knockdown severely impaired the soft agar colony formation of Hep293TT and HUH-6 cells, indicating that FGF19 is necessary for anchorage-independent growth of hepatoblastoma.

These results suggest that hepatoblastoma is dependent on FGF19, which functions as an autocrine growth factor (Figure 2J).

### LY2874455, an inhibitor of FGF receptor kinase, suppresses hepatoblastoma growth

**Figure 3:**
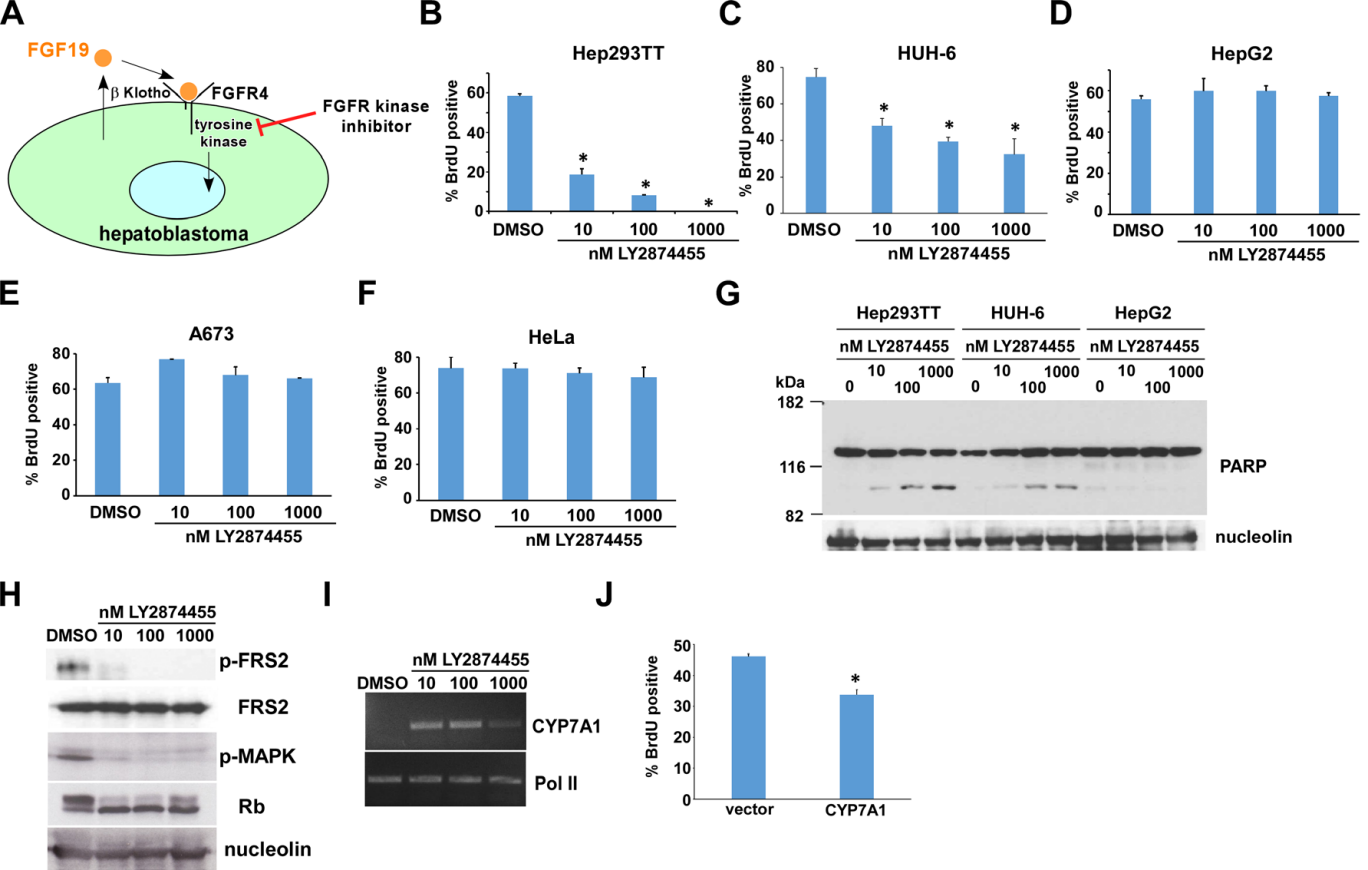
FGF receptor kinase inhibitor LY2874455 suppresses hepatoblastoma proliferation **A.** Suppression of FGF19 signaling by FGFR kinase inhibitor. FGF19 binds to FGFR4 and β-Klotho and stimulates the kinase activity of FGFR4. Therefore, FGF19 signaling can be blocked by pharmacological inhibitors of FGFR kinase activity. **B.** - **F.** LY2874455 inhibits the proliferation of Hep293TT and HUH-6 hepatoblastoma cells, but not HepG2, A673, or HeLa cells. Hep293TT, HUH-6, HepG2, A673, and HeLa cells were treated with DMSO (vehicle) or indicated concentrations of LY2874455 for 24 hours. Cell proliferation was assessed by BrdU incorporation. Asterisks denote *p* < 0.05 compared with DMSO treatment. **G.** LY2874455 induces apoptosis in Hep293TT and HUH-6, but not HepG2 cells. Hep293TT, HUH-6, and HepG2 cells were treated with DMSO (vehicle) or indicated concentrations of LY2874455 for 24 hours. Induction of apoptosis was assessed by immunoblotting for the cleavage of PARP. Nucleolin serves as a loading control. **H.** Dephosphorylation of FRS2, inhibition of MAP kinase, and activation of Rb upon LY2874455 treatment of Hep293TT cells. Hep293TT cells were treated with DMSO (vehicle) or indicated concentrations of LY2874455 for 24 hours. Whole cell lysates were prepared, and immunoblotting was performed using antibodies against phospho-FRS2 (Tyr196), FRS2, phospho-MAPK, Rb, and nucleolin (loading control). **I.** De-silencing of CYP7A1 by LY2874455 treatment of Hep293TT cells. Hep293TT cells were treated with DMSO (vehicle) or indicated concentrations of LY2874455 for 24 hours. Total RNA was isolated and RT-PCR analysis was performed for CYP7A1 and RNA polymerase II (loading control). **J.** CYP7A1 inhibits Hep293TT cell proliferation. Hep293TT cells were infected with lentivirus expressing CYP7A1 or empty vector virus. Four days after infection, cell proliferation was assessed by BrdU incorporation. An asterisk denotes *p* < 0.05 compared with vector.

FGF19 binds to FGF receptor 4 (FGFR4) and its co-receptor β-Klotho (KLB) and transmits signals by stimulating the tyrosine kinase activity of FGFR4 ([10, 11]; Figure 3A). Therefore, FGF19 signaling can be blocked by pharmacological inhibitors of FGFR tyrosine kinase activity such as LY2874455 [12, 13]. We found that blocking FGF19 signaling by LY2874455 for 24 hours results in significant inhibition of Hep293TT and HUH-6 hepatoblastoma cell proliferation (Figure 3B and 3C). In contrast, HepG2 cells, A673 Ewing sarcoma cells, and HeLa cervical cancer cells were not growth inhibited by LY2874455 (Figure 3D - 3F). In addition to proliferation inhibition, LY2874455 treatment also induced apoptosis in Hep293TT and HUH-6 cells, but not HepG2 cells (Figure 3G).

**Figure 4:**
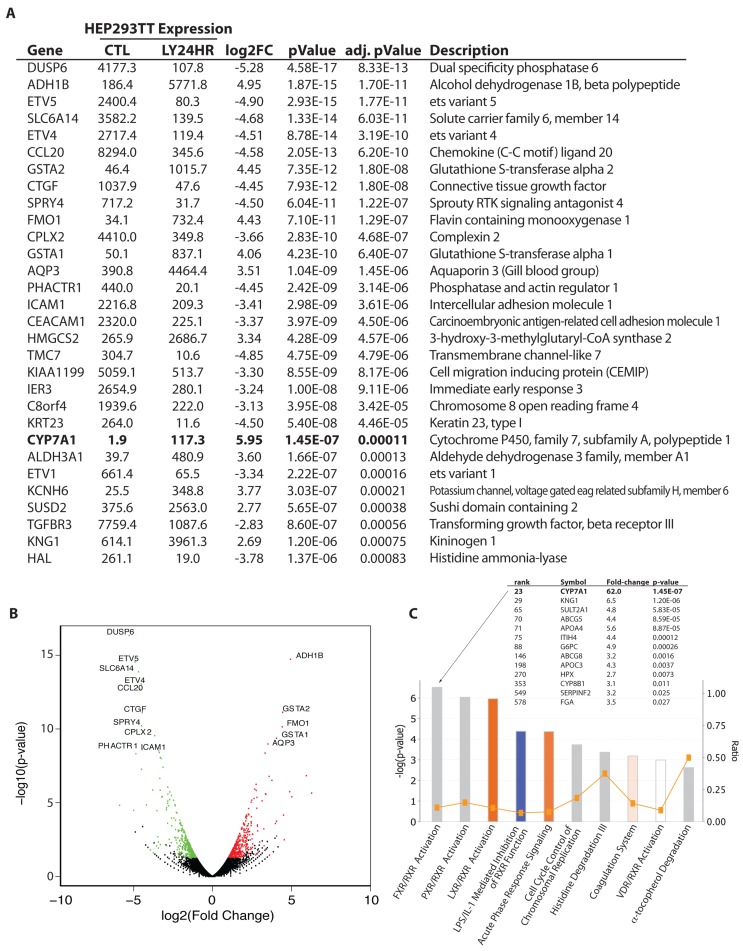
Gene expression changes in LY2874455-treated Hep293TT cells Hep293TT cells were treated with DMSO (vehicle) or 10 nM LY2874455 for 24 hours, total RNA was isolated, and global gene expression was analyzed by RNA-sequencing. Differential gene expression analysis was carried out using DESeq software with sequence read counts for each gene evaluated using HTSeq (see Materials & Methods). **A.** Thirty genes whose adjusted *p*-value < 0.05 (Benjamini-Hochberg correction for multiple test), absolute log2 fold-change > 1, average expression level of control and LY2874455-treated cells > 10, and RPKM > 1 are listed. 802 genes with fold-change > 2 (or absolute log2 fold-change > 1) were selected and submitted to DAVID (see Materials & Methods) for Gene Ontology enrichment analysis. Read counts listed in the table are normalized read counts provided by DESeq. **B.** Volcano plot of all genes, with upregulated genes marked in red and downregulated genes in green. **C.** The Ingenuity Pathway Analysis identified FXR/RXR activation in LY2874455-treated Hep293TT cells.

LY2874455 treatment resulted in dephosphorylation of FRS2, an adaptor protein phosphorylated by FGF19 - FGFR4 signaling, inhibition (dephosphorylation) of MAP kinase, and activation (dephosphorylation) of Rb in Hep293TT cells (Figure 3H), which is consistent with the proliferation arrest induced by LY2874455 (Figure 3B). To further dissect the impact of LY2874455 treatment on hepatoblastoma, global mRNA expression changes were analyzed by RNA-sequencing (Figure 4 and Supplementary Table S3; Figure 4A, list of top 30 genes; Figure 4B, volcano plot of all genes; Figure 4C, the Ingenuity Pathway Analysis). One of the high-scoring gene expression changes induced by LY2874455 was the dramatic upregulation of CYP7A1 (Figure 4A, in bold), an enzyme which catalyzes the first and rate-limiting step of classical bile acid synthesis pathway. FGF19 signaling is known to suppress bile acid biosynthesis through transcriptional repression of CYP7A1 [14, 15]. Therefore, CYP7A1 upregulation by LY2874455 suggests that blocking FGF19 signaling resulted in de-silencing of CYP7A1 and bile acid biosynthesis. Furthermore, the Ingenuity Pathway Analysis identified “FXR/RXR activation” as the top pathway altered by LY2874455 treatment (Figure 4C), suggesting that de-silencing of CYP7A1 and bile acid biosynthesis by LY2874455 resulted in stimulation of transcription mediated by FXR, a nuclear hormone receptor that is activated by its ligands, bile acids [16-18]. De-silencing of CYP7A1 by LY2874455 treatment was verified by RT-PCR analysis (Figure 3I). Interestingly, restoration of CYP7A1 expression in Hep293TT cells by a lentiviral vector resulted in modest, but significant inhibition of Hep293TT cell proliferation (Figure 3J), suggesting that bile acid biosynthesis makes a negative contribution to Hep293TT cell proliferation. CYP7A1 was previously shown to inhibit liver cell proliferation upon partial hepatectomy [19].

### The high expression of FGF19 signaling pathway components as well as the low expression of FGF19 signaling repression targets correlates with the aggressiveness of hepatoblastoma

Based on gene expression profiling, Cairo *et al*., identified two hepatoblastoma sub-types [20]: C1-type, a less aggressive sub-type with features of later developmental stage, and C2-type, a more aggressive sub-type with features of earlier developmental stage. We have recently conducted a comprehensive genomic and transcriptomic analysis of hepatoblastoma tumor samples derived from 62 patients and proposed a new classification of hepatoblastoma sub-types, HB1, HB2, and HB3 (the study will be published elsewhere). HB1 largely corresponds to C1-type described by Cairo *et al.,* while C2-type was sub-divided into HB2 and HB3 based on gene expression profiles, DNA copy number variation, and clinical parameters. HB1 tumors are the least aggressive and the HB3 tumors are the most aggressive while HB2 tumors display intermediate aggressiveness. Using this dataset, we analyzed the expression of FGF19 and its two receptor subunits, FGFR4 and β-Klotho (KLB), in hepatoblastoma tumors and normal liver tissues.

**Figure 5:**
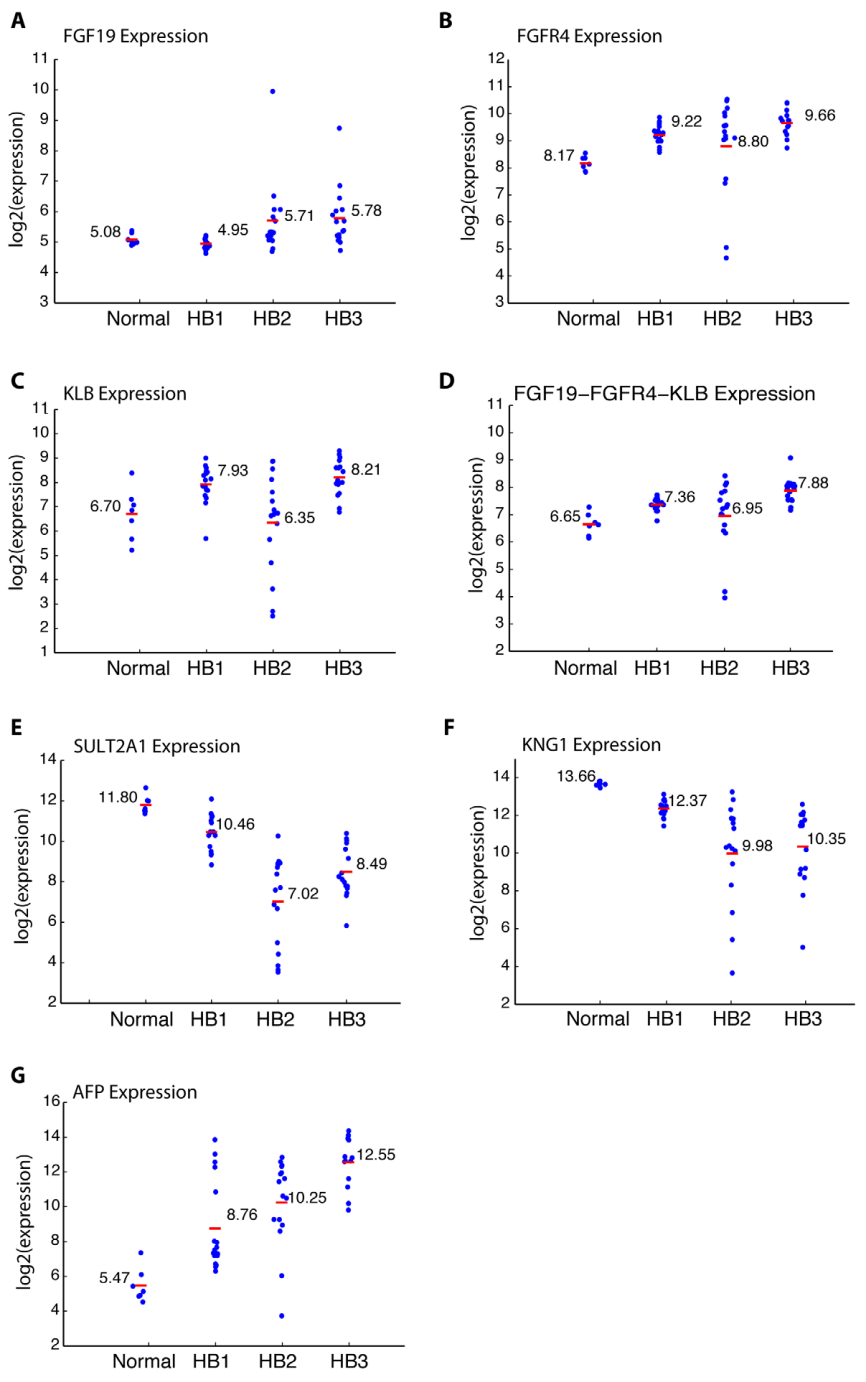
Expression of FGF19, FGFR4, β-Klotho (KLB), SULT2A1, KNG1, and α-fetoprotein (AFP) in HB1, HB2, and HB3 hepatoblastoma tumors and normal liver samples **A.** Expression of FGF19. **B.** Expression of FGFR4. **C.** Expression of β-Klotho (KLB). **D.** Combined expression of FGF19, FGFR4, and β-Klotho (KLB). **E.** Expression of SULT2A1. **F.** Expression of KNG1. **G.** Expression of α-fetoprotein (AFP).

As shown in Figure 5A, we observed a trend toward higher FGF19 expression in aggressive tumors (HB2 *vs*. HB1, *p* = 0.017; HB3 *vs*. HB1, *p* = 0.002). FGFR4 expression was highest in HB3 tumors followed by HB1 tumors (Figure 5B). Similarly, β-Klotho (KLB) expression was highest in HB3 tumors followed by HB1 tumors (Figure 5C). Interestingly, HB2 tumors displayed relatively high FGF19 expression (Figure 5A), but low FGFR4 expression (Figure 5B) and low β-Klotho expression (Figure 5C), which is distinct from HB3 tumors, further supporting the sub-division of C2-type tumors into HB2 and HB3. Using a combination of FGF19, FGFR4, and β-Klotho gene expression (Figure 5D), HB3 tumors could be distinguished from HB1 tumors and HB2 tumors (HB3 *vs*. HB1, *p* = 0; HB3 *vs*. HB2, *p* = 0.01). These results suggest that the expression of FGF19 and its receptor subunits correlates with the grade of hepatoblastoma tumors.

We also analyzed the expression of two FXR target genes, SULT2A1 and KNG1, which were de-silenced by LY2874455 treatment in Hep293TT cells (Figure 4C). As shown in Figure 5E and F, both SULT2A1 and KNG1 displayed significantly lower expression in higher grade tumors, suggesting that the intensity of FGF19 signaling correlates with the aggressiveness of hepatoblastoma. Alpha-fetoprotein (AFP), a marker of hepatoblastoma aggressiveness, displayed higher expression in aggressive tumors as expected (Figure 5G).

**Figure 6:**
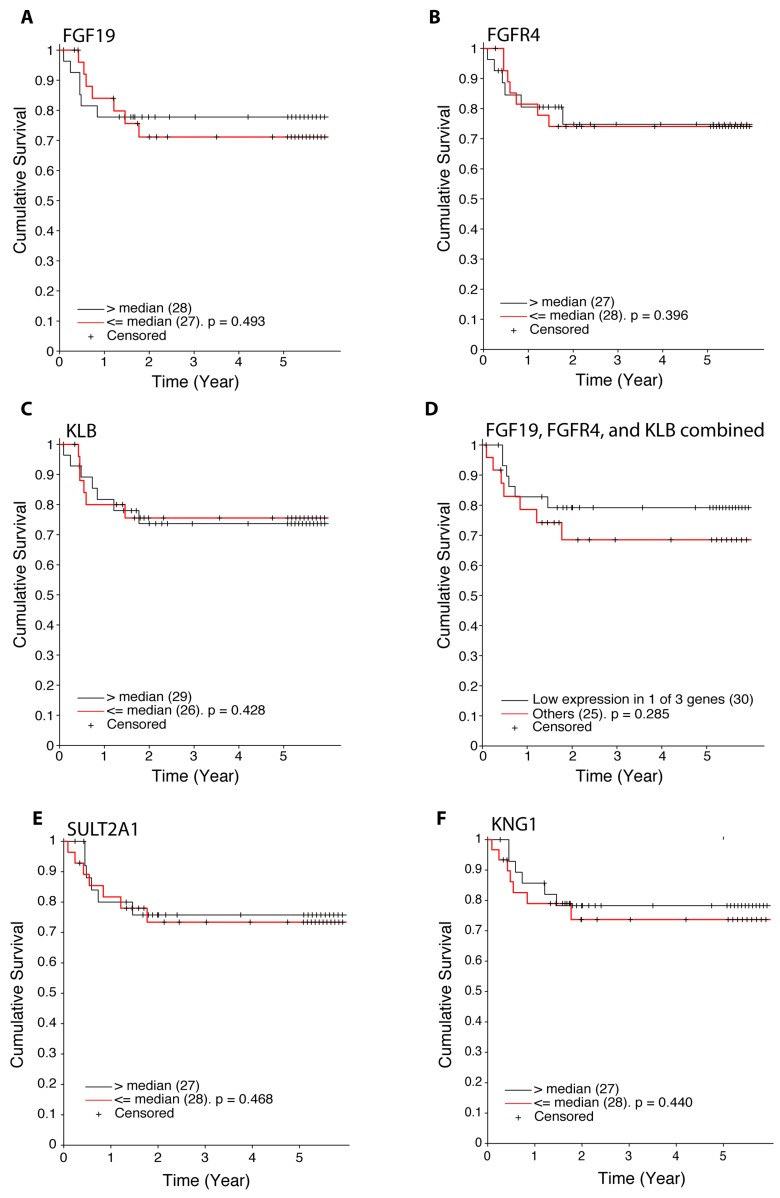
Expression of FGF19, FGFR4, β-Klotho, SULT2A1, and KNG1 in hepatoblastoma tumors and patient survival **A.** Kaplan-Meier survival analysis based on the expression of FGF19. Black and red lines indicate the cumulative survival of hepatoblastoma patients with high (top 50%) and low (bottom 50%) expression of *FGF19.* Significance between the top and bottom 50% was determined by log-rank test. Patients are censored (marked in the figure with “+”) if a patient withdraws from a study, is lost to follow-up, or is alive at last follow-up visit or beyond 5 years. **B.** Kaplan-Meier survival analysis based on the expression of FGFR4. Black and red lines indicate the cumulative survival of hepatoblastoma patients with high (top 50%) and low (bottom 50%) expression of *FGFR4.* Significance between the top and bottom 50% was determined by log-rank test. **C.** Kaplan-Meier survival analysis based on the expression of β-Klotho. Black and red lines indicate the cumulative survival of hepatoblastoma patients with high (top 50%) and low (bottom 50%) expression of β-Klotho. Significance between the top and bottom 50% was determined by log-rank test. **D.** Kaplan-Meier survival analysis based on the combined expression of FGF19, FGFR4, and β-Klotho. To select samples with low expression of the three genes (FGF19, FGFR4 and KLB), we required samples with at least one gene whose expression level is below the 25^th^ percentile (a total of 30 samples). Black and red lines indicate the cumulative survival of hepatoblastoma patients with low expression of FGF19, FGFR4, and β-Klotho (*n* = 30) and the remaining patients (*n* = 25). Significance between the two groups was determined by log-rank test. **E.** Kaplan-Meier survival analysis based on the expression of SULT2A1. Black and red lines indicate the cumulative survival of hepatoblastoma patients with high (top 50%) and low (bottom 50%) expression of *SULT2A1.* Significance between the top and bottom 50% was determined by log-rank test. **F.** Kaplan-Meier survival analysis based on the expression of KNG1. Black and red lines indicate the cumulative survival of hepatoblastoma patients with high (top 50%) and low (bottom 50%) expression of *KNG1.* Significance between the top and bottom 50% was determined by log-rank test.

Employing the expression of FGF19, FGFR4, β-Klotho, or the cumulative expression of FGF19, FGFR4, and β-Klotho, we calculated the overall survival of hepatoblastoma patients (Figure 6A - 6D). Patients with low expression in at least one of FGF19, FGFR4, and β-Klotho displayed a modest, although not statistically significant increase in overall survival (Figure 6D, p = 0.285 by log-rank test). We also analyzed the hepatoblastoma patient survival with respect to the expression levels of SULT2A1 and KNG1, the two FGF19 - FXR signaling targets, and found that patients with low expression of SULT2A1 or KNG1 display a slight, but statistically insignificant decrease in overall survival (Figure 6E and 6F). Patient survival is affected by many factors and hence it is not surprising that the expression levels of the components or downstream targets of a single pathway do not show strong correlation with patient survival. These results suggest that the expression of FGF19, FGFR4, β-Klotho, SULT2A1, and KNG1 by themselves, although may have functional significance, at this time do not appear to be useful as prognosis markers. Additional future studies, integrating with other clinical and molecular features and larger sample sizes, may further clarify if the expression of these genes plays a role in prognosis

In this study, we demonstrated that childhood hepatoblastoma is dependent on autocrine FGF19 signaling and that the high expression of FGF19 signaling pathway components as well as the low expression of FGF19 signaling repression targets correlates with the aggressiveness of hepatoblastoma. A previous genomic analysis of adult hepatocellular carcinoma identified FGF19 gene amplification in about 15% of cases and demonstrated that FGF19 functions as oncogenic driver in this cancer [7]. In childhood hepatoblastoma, FGF19 gene amplification does not appear to be as prevalent as in adult hepatocellular carcinoma (FGF19 gene amplification observed in 2/40 (5%) of hepatoblastoma tumors analyzed). However, the higher mRNA expression of FGF19 and its receptor subunits (FGFR4 and β-Klotho) as well as the lower expression of two FGF19 signaling repression targets (SULT2A1 and KNG1) correlated with higher tumor grade of hepatoblastoma, suggesting that FGF19 signaling promotes hepatoblastomagenesis. It will be important in the future to test the feasibility of targeting FGF19 signaling as therapy for childhood hepatoblastoma, particularly the more aggressive subtypes which are most in need of novel therapies.

## MATERIALS AND METHODS

### Cell culture and reagents

Hep293TT [4] and HUH-6 ([21]; purchased from Japanese Collection of Research Bioresources Cell Bank) hepatoblastoma cell lines were cultured in RPMI 1640 supplemented with 10% fetal calf serum, 25mM HEPES, and 1mM sodium pyruvate. 293T cells were cultured in DMEM supplemented with 10% calf serum. HepG2, A673, and HeLa cells were cultured in DMEM supplemented with 10% fetal calf serum. Calcium phosphate co-precipitation was used for plasmid DNA transfection. LY2874455 was purchased from Axon Medchem. Lentiviruses were prepared by transfection in 293T cells following System Biosciences' protocol and the cells infected with lentiviruses were selected with 2 μg/ml puromycin for 48 hours as described [22, 23]. The target sequences for shRNAs are as follows: human FGF19 shRNA-1, ACTTGTCTGATCATAACATTG; human FGF19 shRNA-2, GCTTTCTTCCACTCTCTCATT; and control shRNA, CCTAAGGTTAAGTCGCCCTCG. CYP7A1 cDNA was cloned into pCDH1 lentiviral vector (System Biosciences).

### Protein sample preparation and mass spectrometry

Hep293TT cells and HepG2 cells were washed six times with PBS and the medium was changed to DMEM without serum. Cells were cultured for 24 hours and the culture supernatant was harvested. The supernatant was centrifuged, filtered through a 0.45 μm filter (Millipore), and concentrated using a 3,000 Dalton cut-off Amicon Ultra Centrifugal Filter Units (Millipore). The proteins in each sample were fractionated by SDS-PAGE and visualized by Coomassie blue. Each gel lane was divided into six slices, and the proteins in each slice were digested *in situ* with trypsin (Promega modified) in 40 mM NH_4_HCO_3_ overnight at 37°C. The resulting tryptic peptides were analyzed by HPLC-ESI-tandem mass spectrometry (HPLC-ESI-MS/MS) on a Thermo Fisher LTQ Orbitrap Velos mass spectrometer fitted with a New Objective Digital PicoView 550 NanoESI source. On-line HPLC separation of the digests was accomplished with an Eksigent/AB Sciex NanoLC-Ultra 2-D HPLC system: column, PicoFrit™ (New Objective; 75 μm i.d.) packed to 15 cm with C18 adsorbent (Vydac; 218MSB5 5 μm, 300 Å); mobile phase A, 0.5% acetic acid (HAc)/0.005% trifluoroacetic acid (TFA); mobile phase B, 90% ACN/0.5% HAc/0.005% TFA; gradient 2 to 42% B in 120 min; flow rate, 0.4 μl/min. Precursor ions were acquired in the Orbitrap in centroid mode at 60,000 resolution *(m/z* 400); data-dependent collision-induced dissociation (CID) spectra of the 10 most intense ions in the precursor scan above a threshold of 3,000 were acquired at the same time in the linear trap (isolation window for MS/MS, 3; relative collision energy, 30). Ions with a 1+ or unassigned charge state were not fragmented. Dynamic exclusion settings were: repeat count, 1; repeat duration, 30 sec; exclusion list size, 500; exclusion duration, 30 sec.

### Mass spectrometry data analysis

The Xcalibur raw files were converted to mzXML format using ReAdW (http://tools.proteomecenter.org/wiki/index.php?title=Software:ReAdW) and were searched against the IPI human protein database (v 3.24; 66,923 protein entries) using X! TANDEM CYCLONE TPP (2011.12.01.1 - LabKey, Insilicos, ISB). Methionine oxidation was considered as a variable modification in all searches. Up to one missed tryptic cleavage was allowed. The X! Tandem search results were analyzed by the Trans-Proteomic Pipeline [24] version 4.3. Peptide/protein identifications were validated by Peptide/ProteinProphet [25, 26]. A ProteinProphet score of 0.9 was used as a cutoff, which corresponded to false identification rates of 0.8 % and 0.7 % in the Hep293TT and HepG2 datasets, respectively.

### Immunoblotting, immunofluorescence, ELISA, and antibodies

Immunoblotting and immunofluorescence were performed as described [22, 23]. FGF19 protein levels in the medium were determined using human FGF-19 Quantikine ELISA Kit (R&D Systems). The following antibodies were used: mouse monoclonal anti-BrdU (BD Pharmingen); mouse monoclonal anti-Ki-67 (BD Pharmingen); mouse monoclonal anti-nucleolin (C23, Santa Cruz Biotechnology); rabbit polyclonal anti-PARP (#9542, Cell Signaling); mouse monoclonal anti-phospho-ERK (E4, Santa Cruz Biotechnology); rabbit polyclonal anti-phospho-FRS2 (Tyr196) (#3864, Cell Signaling); mouse monoclonal anti-FRS2 (MAB4069, R&D Systems); and mouse monoclonal anti-RB (G3-245, BD Pharmingen).

### RT-PCR

Total cellular RNA was isolated using TRIzol reagent (Invitrogen) and RT-PCR was performed as described previously [22, 23]. The following primers were used: RNA polymerase II (Pol II) 5′ primer, GGATGACCTGACTCACAAACTG, 3′ primer, GCCCAGACTTCTGCATGG; FGF19 5′ primer, AGATCAAGGCAGTCGCTCTG, 3′ primer AAAGCACAGTCTTCCTCCGA; CYP7A1 5′ primer, CAGAACTGAATGACCTGCCA, 3′ primer, GGTGCAAAGTGAAATCCTCC. The quantitative real-time RT-PCR was performed using GoTaq^®^ qPCR Master Mix (Promega) and 7500 Real-Time PCR System (Applied Biosystems).

### Antibody neutralization assay

Hep293TT and HUH-6 cells were seeded on glass coverslips. Cultures were treated with 10 μg/ml normal mouse IgG (sc-2025, Santa Cruz Biotechnology) or 5 μg/ml and 10 μg/ml anti-FGF19 antibody (117611, R&D systems) for 24 hours. Cells were subsequently evaluated for Ki-67 staining by immunofluorescence microscopy as described [22, 23].

### Soft agar colony formation assay

Hep293TT and HUH-6 cells were infected with lentiviruses expressing shRNAs against FGF19 or control shRNA and were selected with 2 μg/ml puromycin. Four days after infection, 1×10^3^ cells were plated in soft agar. The soft agar cultures were comprised of two layers: a base layer (2 ml/well in a 6-well plate; RPMI1640, 10% fetal calf serum, 25mM HEPES, 1mM sodium pyruvate, and 1.2% agarose) and a cell layer (2 ml/well in a 6-well plate; RPMI1640, 10% fetal calf serum, 25mM HEPES, 1mM sodium pyruvate, and 0.6% agarose). Colonies were grown for one week and counted.

### RNA sequencing

Hep293TT cells were treated with DMSO (vehicle) or 10 nM LY2874455. Twenty-four hours later, total RNA was isolated using TRIzol reagent (Invitrogen). RNA quality was assessed by Bioanalyzer and poly A(+) RNA was isolated by oligo-dT purification and fragmented using divalent cations under elevated temperature. cDNA fragment libraries were synthesized following the TruSeq mRNA-seq Library Preparation protocol (Illumina, San Diego, CA). We obtained 26.2 and 24.4 million sequence reads for control and LY2874455-treated samples, respectively, using Illumina HiSeq system at the Greehey Children's Cancer Research Institute Genome Sequencing Facility, employing a 50bp single-read sequencing protocol.

Sequence reads were first aligned with TopHat [27] to human genome (NCBI GRCh37/UCSC hg19), allowing no more than 2 mismatches in the alignment. After alignment, reads aligned to known transcripts were counted using HTSeq [28]. Expression abundance of each gene was evaluated by a unit of read count and RPKM (read per kilobase of transcript per million reads mapped). Differential gene expression was calculated using DESeq [29] to obtain fold-change, *p*-value, and *p*-value adjusted by Benjamini-Hochberg correction for multiple tests [30]. We selected differentially expressed genes based on the following criteria: 1) fold-change > 2 (and adjusted *p*-value < 0.05) and 2) RPKM > 1. Functional assessment of these differentially expressed genes was performed by using Database for Annotation, Visualization and Integrated Discovery (DAVID, http://david.abcc.ncifcrf.gov/) [31] and Ingenuity Pathway Analysis (IPA, Ingenuity Systems, http://www.ingenuity.com).

### Hepatoblastoma tumor samples, RNA expression analysis, and Kaplan-Meier survival analysis

A total of 62 hepatoblastoma samples (including 7 normal liver tissues from children with similar age group; details of these samples will be published elsewhere) were profiled using Affymetrix's Human Genome U133 plus 2 GeneChip (Affymetrix, Santa Clara, CA), following the manufacturer's protocol. The U133plus2 GeneChip contains 54,675 probe sets designed for the detection of human gene expression. Expression values were calculated using the robust multi-chip average method (RMA; [32]). Combining the clinical parameters, DNA copy number variation, and gene expression similarity, three unique hepatoblastoma groups (HB1, HB2 and HB3) were determined (the study will be published elsewhere). Gene expression differences between these groups for selected genes (FGF19, FGFR4, KLB, SULT2A1, KNG1, and AFP) were evaluated by using two-population Student *t*-test. Kaplan-Meier plots were generated using MATLAB (Mathworks, MA), with significance *p*-values from log-rank tests. To select samples with low expression of three genes (FGF19, FGFR4 and KLB), we required samples with at least one gene whose expression level was below the 25^th^ percentile (a total of 30 samples).

## SUPPLEMENTARY MATERIAL TABLES


